# Concentrations of the serum long-chain omega-3 polyunsaturated fatty acids and hair mercury in men in different apolipoprotein E phenotypes

**DOI:** 10.1007/s10534-025-00677-7

**Published:** 2025-03-14

**Authors:** Unna Fagerholm, Heli E. K. Virtanen, Tomi-Pekka Tuomainen, Jukka T. Salonen, Jyrki K. Virtanen

**Affiliations:** 1 Institute of Public Health and Clinical Nutrition, University of Eastern Finland, P.O. Box 1627, 70211 Kuopio, Finland; 2 Faculty of Medicine, Department of Public Health, University of Helsinki, Helsinki, Finland; 3 MAS-Metabolic Analytical Services Oy, Helsinki, Finland

**Keywords:** Apolipoprotein E, Cohort study, Fish, Long-chain omega-3 fatty acids, Mercury, Polyunsaturated fatty acids

## Abstract

Fish is a source of long-chain omega-3 polyunsaturated fatty acids (LC n-3 PUFA) and methylmercury, a toxic heavy metal, with opposite effects on cardiovascular disease risk and cognitive decline. Besides diet, the apolipoprotein E (APOE) genotype may affect LC n-3 PUFA and mercury concentrations in the body, but the evidence is inconsistent. The subjects were 1159 men aged 42–60 years, examined in 1984–1989. ANCOVA and linear regression were used in the analyses. The mean ± SD concentrations of serum eicosapentaenoic acid (EPA), docosapentaenoic acid (DPA) and docosahexaenoic acid (DHA) were 1.57 ± 0.82, 0.55 ± 0.10 and 2.45 ± 0.75%, respectively. There were no differences in LC n-3 PUFA concentrations between APOE4 carriers and non-carriers (*P*-values ≥ 0.60). The mean ± SD hair mercury concentration was 1.55 ± 1.3 µg/g. The concentrations were slightly higher in APOE4 carriers vs. non-carriers (difference 0.16 µg/g, 95% confidence interval = 0.01–0.32,*P* = 0.04). Overall, fish consumption was associated with higher hair mercury and serum EPA and DHA concentrations, but no differences in the associations were found between APOE4 carriers and non-carriers (*P*-interactions ≥ 0.30). Hair mercury, but not serum LC n-3 PUFA concentrations, were higher in APOE4 carriers vs. non-carriers. However, as no differences were found in the associations of fish intake with LC n-3 PUFA and mercury concentrations, the results could be due to differences in mercury accumulation.

## Introduction

Seafood is the major dietary source of the long-chain omega-3 polyunsaturated fatty acids (LC n-3 PUFAs), including eicosapentaenoic acid (EPA), docosapentaenoic acid (DPA) and docosahexaenoic acid (DHA), which play a key role in multiple functions in the body. In a recent meta-analysis of 38 randomized controlled trials, higher intake of LC n-3 PUFAs reduced overall cardiovascular disease (CVD) risk and improved cardiovascular outcomes (Khan et al. 2021). Prospective cohort studies show similar beneficial associations with risk of all-cause and cause-specific mortality, including CVD and cancer mortality (Harris et al. 2021). Also, low LC n-3 PUFA intake and plasma concentrations have been associated with increased risk of cognitive decline and developing dementia and Alzheimer’s disease (Troesch et al. 2020).

However, due to the bioaccumulation and biomagnification in the food-chain, especially old, predatory fish such as tuna and pike may have a high content of methylmercury, a neurotoxicant. Methylmercury can cross lipid membranes and reach the central nervous system due to its high solubility in lipids (Arrifano et al. 2018). A developing brain is particularly vulnerable to methylmercury toxicity, and exposure to high concentrations of mercury can lead to neurodevelopmental disorders or even fetal deaths (Castoldi et al. 2003), as well as neurodegenerative disorders such as dementia and Alzheimer’s disease later in life (Chin-Chan et al. 2015). Even lower exposure levels to methylmercury from a diet high in seafood have been associated with atherosclerosis progression and risk of CVD (Salonen et al. 2000; Virtanen et al. 2005).

Besides diet, genetic factors, among multiple other mechanisms, can affect LC n-3 PUFA and mercury concentrations in the body. The most studied genetic factor is Apolipoprotein E (APOE) allele epsilon ε4 (APOE4), which is known to increase the risk of CVD (Marais 2019) and cognitive decline (Huang et al. 2019). Some studies (Fisk et al. 2018; Leskinen et al. 2022; Plourde et al. 2009; Samieri et al. 2013), but not all (Chouinard-Watkins et al. 2013; Conway et al. 2014; Liang et al. 2013; Whalley et al. 2008), have shown that those who carry the APOE ε4 allele have lower levels of plasma LC n-3 PUFA compared to those with the ε2 or ε3 alleles. It has also been speculated that mercury accumulation in the body may be greater in the APOE4 carriers than in those with the APOE3 and APOE2 genotypes (de Arrifano et al. 2018a). One reason for this could be the presence (in APOE2 and APOE3) or absence (in APOE4) of the amino acid cysteine in the apolipoprotein, because of the affinity of mercury to the sulfhydryl groups of cysteine (de Arrifano et al. 2018a). This could lead to impaired removal of mercury from the body by the APOE4-containing lipoproteins and potentially contribute to the increased risk of CVD and cognitive disorders in those with the APOE4 genotype. However, there is no strong evidence for the differences in the binding capacity of the different APOE isoforms (Berntsson et al. 2022) and, overall, there is currently very little research evidence of the differences in mercury accumulation in different APOE genotypes (de Arrafiano et al. 2018b).

As the evidence remains scarce and inconsistent, the aim of this study was to evaluate differences in the serum LC n-3 PUFA and hair mercury concentrations in different APOE phenotypes in men. We also evaluated whether the associations of fish intake with these concentrations differ between the APOE phenotypes.

## Methods

### Study population

The subjects were participants in the Kuopio Ischaemic Heart Disease Risk Factor Study (KIHD), an on-going population-based prospective cohort study, which was originally designed to investigate risk factors for CVD, atherosclerosis, and other chronic diseases in men from the city of Kuopio and neighboring communities in Eastern Finland (Salonen 1988). A total of 2682 men who were 42, 48, 54, or 60 years old at baseline (82.9% of those eligible) were recruited in two cohorts. The first cohort consisted of 1166 men who were 54 years old, enrolled between 1984 and 1986, and the second cohort included 1516 men who were 42, 48, 54 or 60 years of age, enrolled between 1986 and 1989. The baseline characteristics of the entire study population have been described previously (Salonen 1988, 1991). The KIHD study protocol was approved by the Research Ethics Committee of the University of Kuopio and complied with the Declaration of Helsinki. All subjects gave their written informed consent for participation.

The APOE phenotype was determined from blood samples from a total of 1340 men. The men with data on APOE phenotype were in general healthier and had more favorable lifestyle and dietary habits than those men whose phenotype data was not assessed (Virtanen et al. 2016). Of the 1340 men, those with missing data on serum fatty acids (n = 56), hair mercury concentrations (n = 52), or fish intake (n = 11) were excluded. After further excluding the outliers in the hair mercury concentrations (values higher or lower than 1.5 times the interquartile range, n = 62), there were 1159 men in the analyses. There were no outlier values in the serum fatty acid concentrations.

### Serum fatty acid and hair mercury measurements

At the baseline examinations, venous blood samples were collected between 8 and 10 AM. Subjects were instructed to abstain from ingesting alcohol for 3 days and from smoking and eating for 12 h prior to giving the sample. Serum fatty acids were determined in 1991 from samples that had been stored at − 80°. Fatty acids were chromatographed in an NB-351 capillary column (HNU-Nordion) using a Hewlett-Packard 5890 Series II GC (Hewlett-Packard Company, since 1999 Agilent Technologies Inc.) with a flame ionisation detector without preseparation (Laaksonen et al. 2002). Serum fatty acids were extracted using chloroform–methanol. The chloroform phase was evaporated and treated with sodium methoxide, which methylated esterified fatty acids. Quantification was carried out with reference standards purchased from NU-Chek Prep Inc. Each analyte had an individual reference standard, and the internal standard was eicosane. Results were obtained in micromoles per litre and presented as proportions of total serum fatty acids. The coefficients of variation (CV%) for repeated measurements of fatty acids were 10.4% for EPA (20:5n-3), 12.7% for DPA (22:5n-3) and 13.3% for DHA (22:6n-3).

Mercury was determined from hair between May 1992 and August 1993 by flow injection analyses, cold vapor atomic absorption spectrometry, and amalgamation (Salonen et al. 1995). Hair samples were processed in a random order at the Department of Chemistry of the University of Kuopio (now University of Eastern Finland, Kuopio campus). All reagents were of analytical grade unless indicated otherwise. All solutions were prepared with ultrapure water with a specific resistivity of 18 MΩ/cm (Millipore). Sample mineralization was performed with a Microwave Digestion System (model MDS-81D, CEM Corp). Dry hair samples (3–50 mg) were treated with a mixture of Suprapur nitric acid and hydrochloric acid. After mineralization, samples were stabilized with potassium permanganate. The mercury in the samples, standards diluted from a stock mercury solution (1000 mg/L, Merck), and quality control materials were determined with the FIAS-200 Flow Injection Analysis and Amalgam System (Bodenseewerk Perkin-Elmer GmbH). Mercury was reduced in the Chemifold of FIAS-200 to atomic mercury vapor with NaBH_4_. Atomic mercury vapor was first carried by a stream of 99.998% argon (AGA) into the Amalgam System and next into the quartz cell in the Perkin-Elmer Zeeman 5000 Spectrometer (Bodenseewerk Perkin-Elmer GmbH), where the quantity of mercury was measured at 253.7 nm. The mean values of mercury content in the UPPS85 hair pool, in the flour, and in the BCR material were 1.10, 5.44, and 1.97 μg/g, and the CV% between sample batches were 7.3, 3.9, and 6.1%, respectively. The CV% for a subject’s hair were 8.6% (n = 48), 7.8% (n = 52), and 7.7% (n = 92) in three periods, during each of which one batch was used.

### Other measurements

Detailed descriptions of the assessments of serum lipids and lipoproteins (Salonen et al. 1992), participants’ medical history and medication use (Salonen et al. 1992), smoking (Salonen et al. 1992), alcohol intake (Salonen et al. 1992), resting blood pressure (Salonen et al. 1992) and physical activity (Lakka et al. 1994) were previously published. Hypertension diagnosis was defined as blood pressure > 140/90 mm Hg at study visit or as the use of antihypertensive medication. Diabetes mellitus was defined as self-reported diabetes or fasting blood glucose of ≥ 6.7 mmol/L (26). Education was assessed in years using a self-administered questionnaire. Body mass index (BMI) was computed as the ratio of weight in kilograms to the square of height in meters, both measured by a study nurse during the study visit. Serum high-sensitivity C-reactive protein (CRP) was measured with an immunometric assay (IMMULITE High Sensitivity CRP Assay; DPC).

The consumption of foods was assessed at baseline with a 4-day guided food record, where 1 day was a weekend day, by using household measures. A picture book with 126 most common foods and drinks consumed in Finland in the 1980s was used to help estimate portion sizes, and instructions were given and completed food records were estimated by a nutritionist. Nutrient intakes were estimated using the NUTRICA 2.5 software (Social Insurance Institution, Helsinki, Finland) (Knuts et al. 1998). The databank of the software is based mainly on Finnish values of nutrient composition of foods (Rastas et al. 1989).

The APOE phenotype was determined from plasma with isoelectric focusing and immunoblotting techniques. Subjects with phenotypes 3/4 or 4/4 were included in the APOE4 group.

### Statistical analysis

The baseline characteristics of the KIHD study population were described using means with standard deviation for continuous variables and percentages for categorical variables. *T*-test and chi-square tests were used to test the differences between the APOE4 carriers and non-carriers. Welch univariate analysis of variance was used to assess unadjusted differences in LC n-3 PUFA and hair mercury concentrations in different APOE phenotypes. Analysis of covariance (ANCOVA) was used to assess multivariable-adjusted differences in the serum LC n-3 PUFA and hair mercury concentrations between the APOE phenotypes. Due to the small number of subjects with APOE2/2, 2/3, 2/4 or 4/4 phenotypes, in these analyses carriers of the allele ε4 (APOE3/4 or 4/4) were compared to the noncarriers. Three different models were used. The first model was unadjusted. The second model was adjusted for age and examination year. The third model further included potential confounders including BMI, fish intake, pack-years of smoking and alcohol intake. The confounders were selected based on factors that are known to affect the serum LC n-3 PUFA or hair mercury concentrations. Relationships of fish intake with hair mercury and LC n-3 PUFA concentrations were estimated by Spearman correlation coefficients and linear regression analysis. All P-values were two-tailed (a = 0.05). All data were analyzed using SPSS 27.0 for Mac (IBM Corp., Armonk, New York, USA).

## Results

### Baseline characteristics

Baseline characteristics of the participants in the whole study population and among the APOE4 carriers and non-carriers are presented in Table 1. The participants of the study were men with the mean age of 52.2 years. For the whole population, the mean concentrations of hair mercury, EPA, DPA and DHA were 1.55 µg/g (SD 1.3), 1.57% (SD 0.82), 0.55% (SD 0.10) and 2.45% (SD 0.75), respectively. Men with the APOE4 phenotype (n = 367, 31.7%) had modestly higher concentrations of serum LDL cholesterol compared to the other men (n = 792, *P* < 0.001) (Table 1). Other variables did not differ statistically significantly between APOE4 carriers and non-carriers.

**Table 1:** Baseline characteristics of the study population

	Whole population (n = 1159)	Apolipoprotein E4 non-carriers (n = 792)	Apolipoprotein E4 carriers (n = 367)	P-value for difference^a^
Age, year	52.2 ± 6.2	52.2 ± 6.3	52.3 ± 6.1	0.685
Education, year	9.1 ± 3.5	9.2 ± 3.5	9.0 ± 3.6	0.394
Leisure-time physical activity, kcal/day	139 ± 154	140 ± 151	137 ± 160	0.717
Body mass index, kg/m^2^	26.7 ± 3.2	26.7 ± 3.2	26.8 ± 3.2	0.561
Current smoker, %	28.5	29.0	27.5	0.274
Diabetes, %	3.9	4.0	3.5	0.167
Hypertension, %	58.3	57.7	59.7	0.401
Lipid-lowering medication use, %	0.7	0.6	0.8	0.127
Blood glucose, mmol/L	4.7 ± 0.9	4.7 ± 0.9	4.7 ± 0.9	0.688
Serum HDL cholesterol, mmol/L	1.3 ± 0.3	1.3 ± 0.3	1.3 ± 0.3	0.094
Serum LDL cholesterol, mmol/L	4.0 ± 1.0	4.0 ± 1.0	4.2 ± 1.0	< 0.001
Serum triglycerides, mmol/L	1.4 ± 0.8	1.4 ± 0.8	1.5 ± 0.9	0.051
Serum C-reactive protein, mg/L	2.3 ± 4.4	2.4 ± 4.0	2.1 ± 5.2	0.291
Alcohol intake, g/week	72 ± 118	69 ± 112	78 ± 129	0.214
*Dietary intakes*				
Energy, kcal/day	2429 ± 591	2421 ± 584	2445 ± 606	0.516
Protein, E%	15.9 ± 2.6	15.9 ± 2.5	16.0 ± 2.6	0.501
Carbohydrates, E%	43.3 ± 6.7	43.4 ± 6.7	43.0 ± 6.7	0.429
Fiber, g/day	25.6 ± 7.6	25.7 ± 7.4	25.4 ± 8.0	0.502
Saturated fatty acids, E%	17.5 ± 4.0	17.5 ± 3.9	17.5 ± 4.0	0.895
Polyunsaturated fatty acids, E%	4.7 ± 1.4	4.7 ± 1.4	4.6 ± 1.4	0.325
Monounsaturated fatty acids, E%	11.8 ± 2.3	11.8 ± 2.3	11.7 ± 2.1	0.417
Trans fatty acids, E%	1.0 ± 0.4	1.0 ± 0.4	1.1 ± 0.4	0.537
Cholesterol, mg/day	391 ± 108	393 ± 110	385 ± 103	0.201
Fruits, berries and vegetables, g/day^b^	261 ± 157	260 ± 156	259 ± 157	0.962
Fish, g/day	43 ± 47	43 ± 49	42 ± 44	0.949
Vegetable margarines, g/day	19 ± 17	18 ± 17	20 ± 17	0.295
Vegetable oils, g/day	2 ± 3	2 ± 3	2 ± 3	0.359

Values are means ± SD or percentages

HDL, high-density lipoprotein; LDL, low-density lipoprotein; E%, percent of energy

^a^From t-test and chi-square test

^b^Excluding potatoes

### Serum long-chain n-3 PUFA, hair mercury and APOE phenotype

The unadjusted mean serum LC n-3 PUFA and hair mercury concentrations in the APOE phenotypes are shown in Table 2. There were no statistically significant differences between the phenotypes.

**Table 2:** Unadjusted concentrations of hair mercury and serum long-chain omega-3 polyunsaturated fatty acids in different apolipoprotein E phenotypes

	Apolipoprotein E phenotype				
	2/2, 2/3, 2/4 (n = 91)	3/3 (n = 701)	3/4 (n = 326)	4/4 (n = 41)	P-value for difference^a^
Hair mercury, μg/g	1.44 (1.17)^b^	1.50 (1.26)	1.63 (1.30)	1.98 (1.56)	0.110
Serum EPA + DPA + DHA, %	4.61 (1.60)	4.57 (1.54)	4.55 (1.57)	4.60 (1.52)	0.989
Serum EPA, %	1.58 (0.90)	1.57 (0.81)	1.56 (0.83)	1.58 (0.77)	0.996
Serum DPA, %	0.55 (0.11)	0.54 (0.10)	0.55 (0.10)	0.56 (0.10)	0.614
Serum DHA, %	2.48 (0.73)	2.56 (0.75)	2.44 (0.75)	2.45 (0.76)	0.986

EPA = eicosapentaenoic acid, DPA = docosapentaenoic acid, DHA = docosahexaenoic acid

^a^From the Welch analysis of variance

^b^Values are mean (standard deviation)

The concentrations of hair mercury and serum LC n-3 PUFA in the APOE4 carriers and non-carriers are shown in Table 3. In the multivariable-adjusted model (Model 2), hair mercury concentrations were, on average, 0.16 µg/g (95% Cl 0.01–0.32) higher in the APOE4 carriers compared to the APOE4 non-carriers (*P* = 0.040). There were no statistically significant differences in the LC n-3 PUFA concentrations between the APOE4 carriers and non-carriers (Table 3).

**Table 3:** Multivariable-adjusted concentrations of hair mercury and serum long-chain omega-3 polyunsaturated fatty acids among apolipoprotein E4 carriers and non-carriers

	APOE4 non-carriers (n = 792)	APOE4 carriers (n = 367)	P-value for difference^a^
Hair mercury, μg/g			
Unadjusted	1.49 (0.05)^b^	1.67 (0.07)	0.031
Model 1	1.50 (0.05)	1.66 (0.07)	0.042
Model 2	1.50 (0.04)	1.66 (0.06)	0.040
Serum EPA, %			
Unadjusted	1.57 (0.03)	1.56 (0.04)	0.885
Model 1	1.57 (0.03)	1.55 (0.04)	0.693
Model 2	1.57 (0.03)	1.55 (0.04)	0.599
Serum DPA, %			
Unadjusted	0.55 (0.004)	0.55 (0.005)	0.779
Model 1	0.55 (0.004)	0.55 (0.005)	0.776
Model 2	0.55 (0.003)	0.55 (0.005)	0.755
Serum DHA, %			
Unadjusted	2.46 (0.03)	2.44 (0.04)	0.779
Model 2	2.46 (0.03)	2.45 (0.04)	0.840
Model 3	2.46 (0.02)	2.44 (0.04)	0.742

Model 1 adjusted for age and examination year

Model 2 adjusted for Model 1, body mass index, fish consumption, alcohol intake and pack-years of smoking

^a^From the analysis of covariance

^b^Values are mean (standard error of the mean)

### Relationship between fish intake and serum long-chain n-3 PUFA and hair mercury concentrations in the APOE4 carriers and non-carriers

In the whole study population, the mean fish intake was 43 (SD 47) g/day and there was no difference in the mean fish intake between the APOE4 carriers and non-carriers (Table 1). Fish intake was associated with higher concentrations of hair mercury and serum EPA and DHA but had no association with the serum DPA concentration (Table 4). There were no differences in the associations between the APOE4 carriers and non-carriers (*P*-interactions ≥ 0.30).

**Table 4:** Association of fish intake with serum long-chain n-3 polyunsaturated fatty acid and hair mercury concentrations in the apolipoprotein E4 carriers and non-carriers

	All subjects (n = 1159)	APOE4 non-carriers (n = 792)	APOE4 carriers (n = 367)	P-value for interaction
Hair mercury, μg/g				
Correlation coefficient	0.31	0.30	0.31	
Change in concentration for each 50 g higher fish intake, mean (95% CI), μg/g^a^	0.36 (0.28–0.43)	0.34 (0.26–0.43)	0.39 (0.24–0.54)	0.66
Serum EPA, %				
Correlation coefficient	0.40	0.44	0.40	
Change in concentration for each 50 g higher fish intake, mean (95% CI), %^a^	0.34 (0.30–0.39)	0.34 (0.29–0.40)	0.33 (0.24–0.42)	0.86
Serum DPA, %				
Correlation coefficient	0.22	0.22	0.18	
Change in concentration for each 50 g higher fish intake, mean (95% CI), %^a^	0.03 (0.02–0.03)	0.03 (0.02–0.03)	0.03 (0.02–0.04)	0.53
Serum DHA, %				
Correlation coefficient	0.43	0.47	0.45	
Change in concentration for each 50 g higher fish intake, mean (95% CI), %^a^	0.33 (0.29–0.37)	0.32 (0.29–0.37)	0.37 (0.29–0.44)	0.30

^a^Model adjusted for age, examination year, body mass index, alcohol intake and pack-years of smoking

## Discussion

In this population-based cohort study focusing on middle-aged men in Eastern Finland, there were no differences in serum LC n-3 PUFA concentrations between the APOE4 carriers and non-carriers. However, hair mercury concentrations were slightly higher in those who were APOE4 carriers, despite similar fish consumption compared to the non-carriers.

Previous studies on LC n-3 PUFA concentrations in different APOE phenotypes have shown inconsistent results. Although the APOE phenotype is known to affect lipid metabolism (Rivera-Íñiguez et al. 2023) and in the present study, the APOE4 carriers had higher LDL cholesterol concentrations (Table 1), no differences in LC n-3 PUFA concentrations were found. Corresponding to our results, some observational studies did not report any significant differences in the LC n-3 PUFA concentrations when comparing the APOE phenotypes (Liang et al. 2013; Whalley et al. 2008; Yassine et al. 2016). Furthermore, studies with an experimental setting have shown varying results: similarly to our findings, total circulating n-3 PUFA concentrations after DHA supplementation were not significantly dependent on the APOE genotype (Conway et al. 2014). On the other hand, some studies indicate that those with the APOE4 allele have fewer profound increases in DHA concentrations after EPA and/or DHA supplementation trials, although the mechanisms are yet not clear (Plourde et al. 2009; Samieri et al. 2013). It has been demonstrated that DHA metabolism could be disturbed in APOE4 carriers compared to non-carriers, and that the rate of β-oxidation of DHA is higher in APOE4 carriers than non-carriers, resulting in lower plasma DHA concentrations (Chouinard-Watkins et al. 2013). Some explanations to the inconsistent results could be the differences in study settings (observational versus experimental) and the source of the LC n-3 PUFA (whole fish versus fish oil capsules), as well as the differences in adjusting for potential cofounders.

We observed that APOE4 carriers had slightly higher hair mercury concentrations compared to non-carriers. Furthermore, in the present analysis, as in previous studies (Dolbec et al. 2001; Morris et al. 2016; Packull-McCormick et al. 2022), higher fish intake correlated with higher hair mercury concentrations. However, there were no differences in this relationship between the APOE4 carriers and non-carriers. Similarly, in a study on Amazonian riverine populations, where fish is a main element of the diet, mercury concentrations were correlated with the frequency of fish consumption, but no differences were found between the APOE genotypes (de Arrifano et al. 2018b). They also found that among those with very high exposure to mercury (hair mercury ≥ 10 µg/g), carriers of APOE4 had higher mercury concentrations in hair than APOE2 carriers, but this difference was not observed among subjects with lower hair mercury concentrations. However, unlike in our study, the analyses were not adjusted for potential confounders. Even though the current evidence seems aligned with our results, to our knowledge, only a few studies focusing on fish intake and mercury concentrations have determined the APOE status.

As the association of fish intake with hair mercury concentrations for the APOE4 carriers does not seem to be stronger compared to the non-carriers, there might not be any substantial differences in mercury absorption between the APOE groups. Thus, the APOE genotype may rather affect mercury excretion or elimination from the body. It has been speculated that the structural differences between the APOE genotypes could explain, at least partially, why APOE4 carriers are more susceptible to heavy metal intoxication and certain diseases, such as Alzheimer’s disease (Andreoli and Sprovieri 2017; Arrifano et al. 2018; Birdsall et al. 2010; Godfrey et al. 2003). The APOE isoforms differ from each other at the amino acid level: APOE4 has arginine in positions 112 and 158, while APOE2 has two cysteines and APOE3 one cysteine and one arginine. Cysteine contains a sulfhydryl group capable of binding metal ions (Ajsuvakova et al. 2020). Therefore, it has been hypothesized that APOE4 does not have the same ability to bind metals such as mercury than the isoforms E2 and E3 have, resulting in reduced clearance from the body and accumulation of mercury in the tissues of the APOE4 carriers (de Arrifano et al. 2018a). Berntsson et al. (2022) tested this theory and did not find any significant differences in binding affinities of inorganic mercury ions between the different APOE isoforms, but instead noticed some structural changes, which were mostly pronounced in APOE4 and might therefore refer to the toxicity of mercury. However, these results do not rule out the possibility that other forms of mercury, such as methylmercury, could have different binding properties to the different APOE isoforms (Berntsson et al. 2022). As this is a fairly new area of research, more investigation is needed to clarify the functions of the APOE protein in mercury metabolism.

The strengths of this study include the population-based cohort study design with a large study population and an extensive database of potential confounders. Serum LC n-3 PUFA and hair mercury have both been established to be reliable biomarkers for exposure (Hodson et al. 2008; Branco et al. 2017). The serum LC n-3 PUFA concentrations reflect the intake during the preceding weeks (Hodson et al. 2008), whereas with the hair growth rate of approximately 1 cm (about 0.39 in) per month, the biomarker represents multiple months of exposure depending on the length of the hair analyzed (Branco et al. 2017). There are also other sources of mercury besides methylmercury in fish, such as the inorganic mercury in dental amalgam fillings, which could contribute to the mercury load and e.g., confound the associations between fish consumption and hair mercury content. However, unlike the organic methylmercury, the inorganic mercury does not readily accumulate in hair, making hair mercury content a good biomarker especially for methylmercury exposure (Branco et al. 2017). Potential weaknesses include the study population of only middle-aged Caucasian men, and the results are therefore not directly generalizable to other populations or to women. We did not have data on the consumption of fish with a high mercury content for exploring whether the intake of such fish would differ between the APOE4 carriers and non-carriers, which could possibly explain the difference in hair mercury concentrations. The groups based on the APOE phenotypes differed in size: most of the subjects had the phenotype 3/3 (n = 701), whereas only a small proportion of subjects had the phenotype 4/4 (n = 41). As this could have created bias in the results, we were able to only compare APOE4 carriers and non-carriers.

## Conclusions

In conclusion, no differences in serum LC n-3 PUFA concentrations were found between the APOE4 carriers and non-carriers. In contrast, hair mercury concentrations were slightly higher in those with the APOE4 phenotype. Although fish consumption was associated with higher concentrations of mercury, EPA and DHA, the associations were similar for the APOE4 carriers and non-carriers. Studies in diverse populations are needed to confirm these findings and investigate the impact of the APOE phenotype on LC n-3 PUFA and mercury concentrations further.

## Data Availability

Data described in this manuscript will not be made available, because it contains sensitive personal data of the subjects, which cannot be completely anonymized.
